# Cooperative Schemes for Joint Latency and Energy Consumption Minimization in UAV-MEC Networks †

**DOI:** 10.3390/s25175234

**Published:** 2025-08-22

**Authors:** Ming Cheng, Saifei He, Yijin Pan, Min Lin, Wei-Ping Zhu

**Affiliations:** 1 School of Communications and Information Engineering, Nanjing University of Posts and Telecommunications, Nanjing 210003, China; hsf_0825@163.com (S.H.); linmin@njupt.edu.cn (M.L.); 2 National Mobile Communications Research Laboratory, Southeast University, Nanjing 210096, China; panyj@seu.edu.cn; 3 Department of Electrical and Computer Engineering, Concordia University, Montreal, QC H3G 1M8, Canada; weiping@ece.concordia.ca

**Keywords:** mobile edge computing (MEC), multi-UAV networks, energy consumption, latency, closed-form enhanced multi-armed bandit (CF-MAB), multi-agent proximal policy optimization (MAPPO)

## Abstract

The Internet of Things (IoT) has promoted emerging applications that require massive device collaboration, heavy computation, and stringent latency. Unmanned aerial vehicle (UAV)-assisted mobile edge computing (MEC) systems can provide flexible services for user devices (UDs) with wide coverage. The optimization of both latency and energy consumption remains a critical yet challenging task due to the inherent trade-off between them. Joint association, offloading, and computing resource allocation are essential to achieving satisfying system performance. However, these processes are difficult due to the highly dynamic environment and the exponentially increasing complexity of large-scale networks. To address these challenges, we introduce a carefully designed cost function to balance the latency and the energy consumption, formulate the joint problem into a partially observable Markov decision process, and propose two multi-agent deep-reinforcement-learning-based schemes to tackle the long-term problem. Specifically, the multi-agent proximal policy optimization (MAPPO)-based scheme uses centralized learning and decentralized execution, while the closed-form enhanced multi-armed bandit (CF-MAB)-based scheme decouples association from offloading and computing resource allocation. In both schemes, UDs act as independent agents that learn from environmental interactions and historic decisions, make decision to maximize its individual reward function, and achieve implicit collaboration through the reward mechanism. The numerical results validate the effectiveness and show the superiority of our proposed schemes. The MAPPO-based scheme enables collaborative agent decisions for high performance in complex dynamic environments, while the CF-MAB-based scheme supports independent rapid response decisions.

## 1. Introduction

Wireless networks have evolved over time into the Internet of Things (IoT), which has promoted emerging applications including autonomous driving, smart cities, virtual reality, and smart healthcare [1,2]. These applications generally rely on the cooperation of a large number of mobile devices, bring exponentially increasing computation load, and require stringent latency standards. However, user devices (UDs) are equipped with limited computing capability and battery capacity, meaning that they struggle to achieve satisfactory performance in these emerging scenarios. Mobile edge computing (MEC) brings high-performance servers to the network edge and provides efficient computing services for UDs [3,4]. MEC is thus a promising technology to address the latency-sensitive and localized processing requirements of future networks.

The terrestrial network infrastructure has a limited coverage, with some dead zones, meaning that it cannot provide sufficient computing and communications services, especially in remote, rural, or disaster-prone areas. Non-terrestrial networks are considered as enablers to provide a reliable and cost-effective solution for continuous and ubiquitous wireless coverage [5,6]. Unmanned aerial vehicles (UAVs) are widely used in current communication networks due to their flexible movement, easy deployment, and line-of-sight (LoS) connections [7,8]. It is effective and economical to deploy swarms of UAVs to form scalable networks. UAVs act as flying edge nodes that can provide computing, caching, and analytic services for UDs on demand. Consequently, UAV-assisted MEC has become an ideal solution to address scenarios where terrestrial infrastructure is unavailable, insufficient, or impractical [9,10].

### 1.1. Related Works

UAV-assisted MEC mainly aims to reduce energy consumption and latency. Effective and efficient resource allocation is key to achieving high performance. Most studies in this area concern energy consumption [11,12,13,14,15,16], latency [17,18,19,20,21,22], or both [23,24,25,26,27,28,29]. Latency and the energy consumption are inherently conflicting, meaning that cost functions are generally introduced as a trade-off. The cost can be defined as the weighted sum of latency and energy consumption [24,27], the minimum value of biased metrics [28], or the specific objective [29]. High system performance can be achieved through the joint optimization of task offloading, communication and computing resource allocation, UAVs’ trajectories, and device association.

Convex optimization methods, such as block coordinate descent (BCD) and successive convex approximation (SCA), are used to solve these non-trivial joint problems. To reduce the energy consumption of a UAV-assisted MEC system, Wang et al. [15] divided the joint optimization of device association, UAVs’ trajectories, task offloading, and resource allocation into three sub-problems. The non-convex trajectory optimization problem is transformed into a convex one through SCA. Then, the BCD method is used to optimize each set of control variables iteratively. To minimize the average response delay in a UAV-assisted MEC system, Zhang et al. [22] also divided the joint optimization problem into three sub-problems of UAV deployment, user access, and task offloading. Then, SCA is used to transform these sub-problems into convex problems and BCD is used to solve them to obtain a near-optimal solution. To minimize the delay, reduce the energy consumption, and maximize the offloaded tasks simultaneously, Sun et al. [28] formulated a multi-objective joint problem, divided it into three sub-problems, and then solved them by using the BCD method. The above studies usually assume that the task arrival rate and channel state are known or can be predicted from historical data. These optimization schemes only aim to obtain short-term optimal solutions based on deterministic conditions.

It is difficult to predict the task arrival and the channel state accurately in many practical scenarios. Buffers are generally used to store the stochastic tasks. Then, the system stability, the queue backlog, and the task completion rate become important and the UAV-assisted MEC system should be investigated from a long-term perspective. The Lyapunov method is one of the most effective solutions to ensure the long-term stability and to maximize the utility [30,31,32,33,34]. Specifically, the Lyapunov method introduces a drift-plus-penalty function that balances the utility and the queue backlog. Then, the original long-term stochastic problem is converted into an online sequence of deterministic problems in each timeslot. These short-term problems minimize the upper bound of the drift-plus-penalty function instead and can be solved by using convex optimization. Zhang et al. [30] minimized the average long-term energy consumption in a UAV-assisted MEC system by jointly optimizing the task offloading, the resource allocation, and the trajectory. Liu et al. [32] investigated the long-term problem in multiple-UAV-assisted MEC systems and took the timeslot scheduling into consideration. Zhao et al. [34] further considered the task caching and migration in multiple-UAV-assisted MEC systems and maximized the long-term average service quantity.

In conventional convex optimization-based and Lyapunov-based methods, the original highly coupled problem is transformed into several approximate convex problems and the local optimal solution is obtained by means of an iterative process and alternative optimization. These methods require well-known information, heavy interaction, and exponentially increasing complexity, meaning that they are unsuitable in practical scenarios with high dynamics or a large scale. Fortunately, deep reinforcement learning (DRL)-based schemes have shown significant advantages in solving these problems with high-dimensional dynamic state space, continuous or discrete action space, and non-linear relationships. DRL-based schemes formulate the joint long-term optimization problem in dynamic environments as a Markov decision process (MDP) [35,36,37,38,39] or partially observable MDP (POMDP) [40,41,42,43,44,45]. Then, one or multiple agents learn from environmental and historic information, update the strategies, and make decisions.

DRL has been applied in UAV-assisted MEC networks [10]. Specifically, to minimize the latency in UAV-assisted MEC systems with caching, Zhang et al. [35] used the actor–critic (AC) architecture to optimize the UAVs’ motion, communication and computing resource allocation, and caching decisions simultaneously. To balance the energy consumption and the latency in multi-UAV-assisted vehicular edge computing systems, Li et al. [36] proposed a double-depth Q-network-based algorithm to optimize the vehicle selection and the resource allocation jointly. Seid et al. [37] proposed a deep deterministic policy gradient (DDPG)-based algorithm to optimize the association, offloading, and computing resource jointly. He et al. [39] further proposed a multi-agent DDPG (MADDPG)-based algorithm to optimize the trajectories of UAVs while power and offloading are determined by conventional optimization. Each agent can only obtain its local observation instead of the global state in most practical scenarios. Yan et al. [40], Liu et al. [41], and Wang et al. [42] formulated the long-term optimization to a POMDP and applied the MADDPG scheme to decide the trajectories of UAVs. Though the above schemes excel in specific scenarios, they show inferior performance regarding adaptability and stability. DQN is limited to discrete actions apace and DDPG is designed for continuous actions space. AC is adaptable to both action spaces but suffers from instable training and poor sample efficiency. Proximal policy optimization (PPO) [46,47,48], which is particularly well-suited for high-dimensional actions space with both continuous and discrete actions, has been successfully applied in UAV-assisted MEC networks [38,43,44,45]. Specifically, Wang et al. [38] proposed a PPO-based algorithm to optimize the continuous movement of UAVs and offloading proportions jointly. Kang et al. [43] further applied the PPO into scenarios with partial observation and proposed a multi-agent PPO (MAPPO)-based scheme to optimize offloading proportion, power control, and computing frequency allocation simultaneously.

The related works are summarized in Table 1.

### 1.2. Motivations and Contributions

UAV-assisted MEC can significantly improve the quality of experience in the IoT. Multi-agent DRL (MADRL)-based methods have shown great potential to improve the efficiency and effectiveness of joint resource allocation. Though MADRL-based methods are generally superior to convex optimization-based and reinforcement learning (RL)-based methods in the performance of their target metrics, their detailed superiority should be further investigated.

This paper investigates a multi-UAV-assisted MEC system in which each UAV is equipped with an MEC server to offer computing services and each UD decides its serving UAV, task offloading proportion, and required computing resources. The network is highly dynamic due to the movement of UAVs and time-varying channel state. We aim to minimize the latency and energy consumption by optimizing the association, task offloading, and computing resource allocation simultaneously. Our previous work [49] proposed an MAB-based distributed scheme to tackle the joint long-term problem. This work significantly extends our previous research with the following contributions:

- We introduce cost functions to balance the latency and energy consumption for both the whole system and individual UDs. Then, we formulate a long-term cost minimization problem that has discrete association constraints and continuous offloading and computing frequency constraints. We further formulate this long-term problem into a POMDP and propose a cooperative multi-agent DRL framework. All UDs are agents and each of them makes its own decision based on its local observation and individual reward function.
- We propose a MAPPO-based scheme that adopts centralized training and decentralized execution to tackle the POMDP. In the training stage, the global observations and the system reward function are used to train the actor and the critic networks for all UDs. In the execution stage, each UD agent uses its local observation and individual reward function for decision-making and network updates. Simulation results validate the MAPPO-based scheme and show its superiority in reducing system cost.
- We decouple the association from the task offloading and the computing resource allocation and propose a lightweight scheme based on closed-form enhanced multi-armed bandit (CF-MAB). In the CF-MAB-based scheme, each UD agent selects its association to maximize its long-term achievable rate and then the optimal offloading and computing resource allocation can be obtained in closed form given the association. Simulation results validate the CF-MAB-based scheme and show its superiority regarding its complexity and task completion rate.

The remainder of this paper is organized as follows. Section 2 describes the system model and formulates the long-term problem. Section 3 presents the MADRL framework, the MAPPO-based scheme, and the MAB-based scheme in detail. Simulation results and discussions are presented in Section 4. Finally, Section 5 provides some concluding remarks.

Some key notations are listed in Table 2.

## 2. System Model

**Figure 1:**
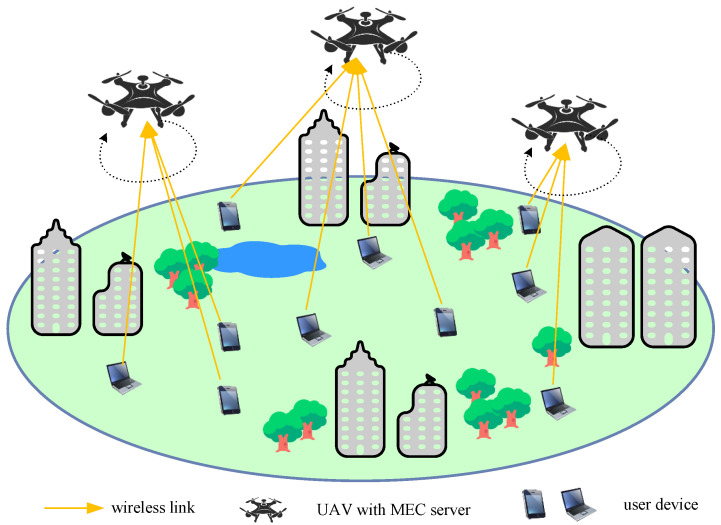
The multi-UAV-assisted MEC system.

Consider a multiple-UAV-assisted MEC system that consists of *N* UAVs and *M* (M>N) UDs as shown in Figure 1. We denote the sets of UDs and UAVs by M={1,2,…,M} and N={1,2,…,N}, respectively. The UDs are randomly located and the UAVs fly along pre-determined trajectories. Each UAV is equipped with an MEC server and provides computing service for UDs. Each UD decides its own association and offloading strategy.

### 2.1. Association

At each timeslot, each UD associates with a suitable UAV. If multiple UDs associate with the same UAV, frequency division multiple access (FDMA) is adopted to avoid interference. The association between UDs and UAVs is indicated by $a_{m,n,t}$. If UD *m* associates with UAV *n* in timeslot *t*, $a_{m,n,t}=1$; otherwise, $a_{m,n,t}=0$. Each UD can only associate with one UAV simultaneously, meaning that we have $\sum_{n\in N}a_{m,n,t}=1$.

### 2.2. Communication Model

The UAVs move continuously and the transmission links between UDs and UAVs can be blocked by buildings or trees. We assume that the state of each transmission link is determined by the environment and the angle of elevation between UDs and UAVs [50]. The transmission link between UD *m* and UAV *n* is line-of-sight (LOS) with probability $p_{m,n}^{LOS}$ and non-line-of-sight (NLOS) with probability $p_{m,n}^{NLOS}\left(t\right)=1-p_{m,n}^{LOS}$.

The channel gain of transmission links is determined by large-scale fading and small-scale fading. The channel gain between UAV *n* and UD *m* is obtained as

(1) $$g_{m,n}=\left\{\begin{array}{c} {\left(\frac{4\pi f_{c}}{c}\right)}^{2}\mu_{L}{d_{m,n}}^{-\beta_{L}}{\left|h_{m,n}^{Rice}\right|}^{2},\;LOS\\ {\left(\frac{4\pi f_{c}}{c}\right)}^{2}\mu_{N}{d_{m,n}}^{-\beta_{N}}{\left|h_{m,n}^{Rayleigh}\right|}^{2},\;NLOS \end{array}\right.$$

where $f_{c}$ is the carrier frequency; *c* is the speed of light; $\beta_{L}$ ($\beta_{N}$) and $\mu_{L}$ ($\mu_{N}$) are the path loss exponent and the attenuation factor for LOS (NLOS) links, respectively; $d_{m,n}$ is the distance between UD *m* and UAV *n*; and $h_{i,j}^{Rice}$ and $h_{i,j}^{Rayleigh}$ are the Rice and Rayleigh fading coefficients, respectively.

Assume that the channel gains are constant during the channel coherent time interval. The signal-to-noise ratio (SNR) between UD *m* and UAV-BS *n* in timeslot *t* is

(2) $$\gamma_{m,n,t}=\frac{Pg_{m,n,t}}{\sigma^{2}}$$

where *P* is the transmission power of the UD, $g_{i,j,t}$ is the channel gain, and $\sigma^{2}$ is the noise power.

$M_{n,t}$ denotes the number of UDs that access UAV *n* in timeslot *t*. Then, the maximum achievable transmission rate between UD *m* and UAV *n* in timeslot *t* is

(3) $$r_{m,n,t}=\frac{B}{M_{n,t}}\log_{2}\left(1+\gamma_{m,n,t}\right)$$

where *B* is the total bandwidth.

### 2.3. Computation Model

At each timeslot, each UD generates its computing tasks with a random size. $D_{m,t}$ denotes the size of tasks generated by UD *m* at timeslot *t*. All UDs adopt the partial offloading policy and determine the offloading proportion. The proportion of tasks that are offloaded to the MEC server is denoted by $\alpha_{m,t}\in \left[0,1\right]$ and $\left(1-\alpha_{m,t}\right)$ is the proportion of tasks that are locally executed.

#### 2.3.1. Edge Computing

The UAV-assisted computing can be divided into three steps: task offloading, edge computing, and result downloading. The UDs first offload their tasks to the serving UAVs and the UAVs compute the received tasks and download the computation results to UDs. The computing results obtained by the MEC server are usually small. The result downloading time is short and can be ignored [17]. The delay in MEC consists of two parts: transmission delay and computation delay.

We neglect the result downloading delay, meaning that the transmission delay equals the task offloading time. The task offloading time from UD *m* to UAV *n* at timeslot *t* can be expressed as

(4) $$\tau_{m,n,t}^{\mathrm{trans}}=\frac{\alpha_{m,t}D_{m,t}}{r_{m,n,t}}$$

The energy consumption observed when UD *m* offloads its task to UAV *n* is given by

(5) $$E_{m,n,t}^{\mathrm{trans}}=P\tau_{m,n,t}^{trans}.$$

The computing time at the MEC server can be expressed as

(6) $$\tau_{m,n,t}^{\mathrm{comp}}=\frac{\alpha_{m,t}D_{m,t}s}{f_{m,n,t}}$$

where *s* is the number of CPU cycles required to process each unit byte and $f_{m,n,t}$ denotes the computing capacity of the UAV *n* allocated to UD *m*. The energy consumption of the UAV *n* to compute the offloading task is given by

(7) $$E_{m,n,t}^{\mathrm{comp}}=\kappa_{1}f_{m,n,t}^{2}\alpha_{m,t}D_{m,t}s$$

where $\kappa_{1}$ is a constant that depends on the CPU of the UAV-assisted MEC server.

#### 2.3.2. Local Computing

The local computation delay of UD *m* at timeslot *t* can be expressed as

(8) $$\tau_{m,t}^{\mathrm{local}}=\frac{\left(1-\alpha_{m,t}\right)D_{m,t}s}{f_{u}}$$

where $f_{u}$ denotes the computing capability of the UD. We assume that all UDs have a predetermined and fixed computing capability.

The energy consumption of the UD computing the local task can be obtained as

(9) $$E_{m,t}^{\mathrm{local}}=\kappa_{2}f_{u}^{2}\left(1-\alpha_{m,t}\right)D_{m,t}s$$

where $\kappa_{2}$ is a constant which depends on the CPU of UD.

### 2.4. System Energy Consumption and Latency

The energy consumption to complete UD *m*’s task in timeslot *t* comes from the local computing and the edge computing, which can be obtained as

(10) $$E_{m,t}=\overset{\mathrm{UD}}{\underset{\mathrm{Local}\;\mathrm{computing}}{\overset{︷}{\underset{︸}{E_{m,t}^{\mathrm{local}}}}}}+\underset{\mathrm{Edge}\;\mathrm{computing}}{\underset{︸}{\sum_{n\in N}a_{m,n,t}\left(\overset{\mathrm{UD}}{\overset{︷}{E_{m,n,t}^{\mathrm{trans}}}}+\overset{\mathrm{UAV}}{\overset{︷}{E_{m,n,t}^{\mathrm{comp}}}}\right)}}.$$

The total energy consumption for tasks computing in timeslot *t* is

(11) $$E_{t}=\sum_{m\in M}E_{m,t}$$

The latency to complete UD *m*’s task in timeslot *t* can be obtained as

(12) $$\tau_{m,t}=\max\left\{\tau_{m,t}^{\mathrm{local}},\sum_{n\in N}a_{m,n,t}(\tau_{m,n,t}^{\mathrm{trans}}+\tau_{m,n,t}^{\mathrm{comp}})\right\}.$$

The system latency is defined as the maximum UD’s latency, which can be obtained as

(13) $$\tau_{t}=\max_{m\in M}\left\{\tau_{m,t}\right\}.$$

### 2.5. Problem Formulation

This paper aims to minimize the energy consumption and the latency simultaneously. We define the system cost in timeslot *t* as the weighted sum of energy consumption and latency [23,24]

(14) $$C_{t}=\lambda E_{t}+\left(1-\lambda \right)\tau_{t}$$

where λ∈[0,1] adjusts the weights. The cost degenerates into energy consumption with λ=1 and latency with λ=0.

Due to the time-varying channel state, UDs should select a suitable serving UAV, make a reasonable offloading decision, and request sufficient computing resources based on the current environment at each timeslot. We jointly optimize the association, offloading proportion, and computing resources allocation to minimize the long-term system cost. The joint optimization problem can be formulated as

(15a) $$\begin{array}{cc} P1:\; & \min_{\left\{a_{m,n,t}\right\},\left\{\alpha_{m,t}\right\},\left\{f_{m,n,t}\right\}}\sum_{t=1}^{\infty }C_{t} \end{array}$$

(15b) $$\begin{array}{cc} s.t.\; & a_{m,n,t}=\left\{0,1\right\},m\in M,n\in N,t\geq 1, \end{array}$$

(15c) $$\begin{array}{cc} & \sum_{n\in N}a_{m,n,t}=1,m\in M,t\geq 1, \end{array}$$

(15d) $$\begin{array}{cc} & \alpha_{m,t}\in \left[0,1\right],m\in M,t\geq 1, \end{array}$$

(15e) $$\begin{array}{cc} & f_{m,n,t}\in \left[0,F_{\max}\right],m\in M,t\geq 1 \end{array}$$

(15f) $$\begin{array}{cc} & \tau_{t}\leq \tau^{\max},t\geq 1 \end{array}$$

where constraints (15b) and (15c) denote that each UD can only associate with one UAV in a timeslot; constraint (15d) is the range of the offloading proportion; constraint (15e) limits the maximum computing resources of the MEC; and constraint (15f) indicates that the latency requirement that cannot exceed the maximum value $\tau^{\max}$. The difficulty of problem P1 lies in the long-term objective and the coupling of association, offloading, and computing resource allocation.

## 3. Multi-Agent DRL-Based Association, Offloading, and Resource Allocation Schemes

Although the trajectories of UAVs can be predetermined, the variation in channel state and the variety in resources allocation make the system highly dynamic. It is challenging to tackle the long-term optimization problem in highly dynamic scenarios, especially for large networks. Fortunately, RL and DRL methods have been applied to solve these sequential decision problems successfully. The multi-agent scheme can further reduce the overheads of information collection and exchange in large networks. Therefore, we adopt the multi-agent RL framework and propose an RL- and a DRL-based scheme. We first introduce the multi-agent RL framework and formulate the long-term problem as a POMDP. Then, we elaborate the MAPPO-based and the CF-MAB-based schemes.

### 3.1. Multi-Agent RL Framework

In the multi-agent RL framework, all UDs are agents. Each agent maintains a stochastic policy to make decisions based on its local observations and then receives its corresponding reward independently. The long-term joint allocation problem can be treated as a POMDP. The policy of agent *m* is denoted by $\pi^{\left(m\right)}\left(a_{t}^{\left(m\right)}|o_{t}^{\left(m\right)}\right)$, where $a_{t}^{\left(m\right)}$ and $o_{t}^{\left(m\right)}$ are the action and observation, respectively. We aim to find a joint policy for all UDs to minimize the long-term cost. The joint policy is the product of individual policies $\pi =\prod_{m=1}^{M}\pi^{\left(m\right)}$.

The *observation* contains the channel gains and the volume of current tasks. The observation of UD *m* at timeslot *t* can be denoted by

(16) $$o_{t}^{\left(m\right)}=\left\{{\left\{g_{m,n,t}\right\}}_{n\in N},D_{m,t}\right\}.$$

We define the global state as the ensemble of observations of all UDs:

(17) $$s_{t}=\left\{{\left\{o_{t}^{\left(m\right)}\right\}}_{m\in M}\right\}.$$

Each UD agent makes an *action* to decide its association, offloading proportion, and computing resource demand based on its policy. The action of UD *m* at timeslot *t* can be denoted by

(18) $$a_{t}^{\left(m\right)}=\left\{{\left\{a_{m,n,t}\right\}}_{n\in N},\alpha_{m,t},f_{m,n_{0},t|a_{m,n_{0},t}=1}\right\}.$$

The joint action of all UDs is denoted by

(19) $$a_{t}=\left\{{\left\{a_{t}^{\left(m\right)}\right\}}_{m\in M}\right\}.$$

After its action, each agent will receive an instantaneous *reward* from the environment. In our multi-agent framework, there is no global information exchange among UD agents, meaning that each UD can only obtain a direct reward based on its own local information, such as the transmission rate $r_{m,n,t}$, energy consumption $E_{m,t}$, and latency $\tau_{m,t}$. The reward of UD *m* after timeslot *t* can be denoted by

(20) $$r_{t}^{\left(m\right)}=\left\{{\left\{r_{m,n,t}\right\}}_{n\in N},E_{m,t},\tau_{m,t}\right\}.$$

Each UD agent can only obtain its local information, meaning that it cannot obtain the system cost. We introduce the individual cost based on its own reward for each UD as

(21) $$C_{m,t}=\lambda E_{m,t}+\left(1-\lambda \right)\tau_{m,t}.$$

Then, each UD agent updates its policy to minimize its individual cost instead.

There is a gap between minimizing the individual cost and minimizing the system cost. We use the substitution based on the fact that the system cost achieves the minimum when all UDs achieve their minimum individual cost synchronously.

All UD agents interact with the environment and other agents through the observation–action–reward process. A good policy is the key to achieving effective learning and efficient cooperation. We propose a MAPPO-based scheme and an MAB-based scheme to find the policies that can minimize the long-term cost.

### 3.2. MAPPO-Based Scheme for Long-Term Consumption Minimization

The MAPPO-based scheme is a multi-agent scheme that uses the AC architecture and the PPO. Specifically, each agent has an actor network and a critic network. The former makes actions and the later evaluates the actions. We first introduce the general MAPPO procedure. Then, we explain the reward functions in centralized training and decentralized execution, respectively. Finally, we summarize the MAPPO-based scheme.

#### 3.2.1. Typical MAPPO Procedure

The actor network and the critic network are parameterized by θ and ϕ, respectively. The expected discounted accumulated reward can be denoted by

(22) $$J\left(\theta \right)=E_{a_{t},s_{t}}[\sum_{t}\gamma^{t}R_{t}],$$

where $R_{t}$ is the instantaneous reward in timeslot *t* and γ is the discount factor. Policy gradient methods maximize the expected reward by repeatedly estimating the gradient on θ. The most commonly used policy gradient has the form

(23) $$\hat{g}={\hat{E}}_{t}\left[\nabla_{\theta }\log\pi_{\theta }\left(a_{t}|s_{t}\right){\hat{A}}_{t}\right]$$

where $\pi_{\theta }\left(a_{t}|s_{t}\right)$ is a stochastic policy, the expectation $\hat{E}\left[\cdot \right]$ indicates the empirical average over a finite batch of samples, and ${\hat{A}}_{t}$ is an estimator of the advantage function at timeslot *t*. We adopt the generalized advantage estimation (GAE) method and use the truncated estimator [51]

(24) $${\hat{A}}_{t}=\delta_{t}+\left(\gamma \lambda_{0}\right)\delta_{t+1}+\cdots +{\left(\gamma \lambda_{0}\right)}^{T-t+1}\delta_{T-1}$$

where *T* is the length of trajectory segment, parameter $\lambda_{0}$ balances the bias and variance, $\delta_{t}=R_{t}+\gamma V\left(s_{t+1}\right)-V\left(s_{t}\right)$, and $V(s_{t})$ is the state-value function.

The actor is trained to maximize the objective function F(θ) as [47]

(25) $$F\left(\theta \right)=\frac{1}{N_{b}M}\sum_{i=1}^{N_{b}}\sum_{m=1}^{M}\left(\min\left\{\kappa_{\theta ,i}^{\left(m\right)}{\hat{A}}_{i}^{\left(m\right)},\mathrm{clip}(\kappa_{\theta ,i}^{\left(m\right)},1-\epsilon ,1+\epsilon ){\hat{A}}_{i}^{\left(m\right)}\right\}+\sigma \nu \left[\pi_{\theta }\left(s_{i}^{\left(n\right)}\right)\right]\right),$$

where $N_{b}$ is the size of the mini-batch; *M* is the number of agents; $\kappa_{\theta ,i}^{\left(m\right)}=\frac{\pi_{\theta ,i}^{\left(m\right)}\left(a_{i}|s_{i}\right)}{\pi_{\theta_{old},i}^{\left(m\right)}\left(a_{i}|s_{i}\right)}$ is the probability ratio between the current policy and the updated policy of agent *m*; ${\hat{A}}_{i}^{\left(n\right)}$ is the advantage that can be estimated with (24); $\mathrm{clip}\;(\kappa_{\theta ,i}^{\left(n\right)},1-\epsilon ,1+\epsilon )$ is a clip function that restricts $\kappa_{\theta ,i}^{\left(m\right)}$ into the interval [1−ϵ,1+ϵ]; σ is the entropy coefficient hyper parameter; and ν is the policy entropy to increase the exploration rate.

The critic network is trained to minimize the objective function [47]

(26) $$L\left(\phi \right)=\frac{1}{N_{b}M}\sum_{i=1}^{N_{b}}\sum_{m=1}^{M}\left(\max\{{\left(V_{\phi }\left(s_{i}^{\left(n\right)}\right)-R_{i}\right)}^{2},{\left(\mathrm{clip}(V_{\phi }\left(s_{i}^{\left(n\right)}\right),V_{\phi_{old}}\left(o_{i}^{\left(n\right)}\right)-\epsilon ,V_{\phi_{old}}\left(o_{i}^{\left(n\right)}\right)+\epsilon )-R_{i}\right)}^{2}\}\right),$$

where $R_{i}$ is the cumulative discounted reward.

The objective functions in (25) and (26) can be optimized through the gradient ascent method. The objective functions are also propagated back to update parameters of the actor and critic by means of the Adam method.

#### 3.2.2. Centralized Training and Decentralized Execution

The MAPPO-based scheme adopts centralized training and decentralized execution. In the training stage, all agents share a centralized critic network that evaluates the actions of all agents. This critic network should know all the global states and all agents’ observations and actions. Therefore, all agents share their local observations and receive the same critic network feedback. However, in the execution stage, each agent only has local observations and actions without coordination. To solve this problem, we use different rewards and gradients for the training and execution stages as follows.

To bridge the system cost and the individual cost, we set two kinds of *reward functions*. Each agent generates its individual reward function based its own cost as

(27) $$R_{m,t}=\frac{1}{C_{m,t}}.$$

Each UD should complete its task in the current timeslot, otherwise it will fail to meet the latency demand. Therefore, the reward function of the whole system should consider the cost of all UDs and the number of UDs that fail. We define the system reward function as

(28) $$R_{M,t}=\left(1-\frac{K_{t}}{M}\right)\sum_{m\in M}R_{m,t}$$

where $K_{t}$ is the number of UDs that fail to complete tasks in timeslot *t* and the term $\left(1-\frac{K_{t}}{M}\right)$ can be a penalty. The overall reward function $R_{M,t}$ is used in training for all agents and the individual reward function $R_{m,t}$ is used in execution for network updates.

#### 3.2.3. MAPPO-Based Algorithm

The MAPPO-based algorithm for UAV-assisted MEC system is summarized in Algorithm 1. $\theta ={\left\{\theta^{\left(m\right)}\right\}}_{m\in M}$ and $\phi ={\left\{\phi^{\left(m\right)}\right\}}_{m\in M}$ are the combinations of parameters of all UD agents, where $\theta^{\left(m\right)}$ and $\phi^{\left(m\right)}$ are the parameters of the actor and the critic of UD agent *m*, respectively. There are some differences in the training stage and the execution stage. In the centralized training stage, the computations in line 11 use the overall reward function $R_{M,t}$, while those in line 15 compute the gradients of objective functions in (25) and (26). In the decentralized execution stage, the computations in line 11 use the individual reward function $R_{M,t}$, while those in line 15 compute the gradients of individual objective functions as

(29) $$F^{\left(m\right)}\left(\theta \right)=\frac{1}{N_{b}}\sum_{i=1}^{N_{b}}\left(\min\left\{\kappa_{\theta ,i}^{\left(m\right)}{\hat{A}}_{i}^{\left(m\right)},\mathrm{clip}(\kappa_{\theta ,i}^{\left(m\right)},1-\epsilon ,1+\epsilon ){\hat{A}}_{i}^{\left(m\right)}\right\}+\sigma \nu \left[\pi_{\theta }\left(s_{i}^{\left(n\right)}\right)\right]\right)$$

and

(30) $$L^{\left(m\right)}\left(\phi \right)=\frac{1}{N_{b}}\sum_{i=1}^{N_{b}}\left(\max\{{\left(V_{\phi }\left(s_{i}^{\left(n\right)}\right)-R_{i}\right)}^{2},{\left(\mathrm{clip}(V_{\phi }\left(s_{i}^{\left(n\right)}\right),V_{\phi_{old}}\left(o_{i}^{\left(n\right)}\right)-\epsilon ,V_{\phi_{old}}\left(o_{i}^{\left(n\right)}\right)+\epsilon )-R_{i}\right)}^{2}\}\right).$$

**Algorithm** **1** MAPPO-Based algorithm1:Initialize parameter θ of the actor network and parameter ϕ of the critic network.2:**for** each episode i=1,…,Ep **do**3:   Initialize experience reply buffer *U*;4:   **for** each timeslot t=1,…,T **do**5:     **for** each UD m∈M **do**6:        Execute action according to $\pi_{\theta_{old}}(a_{t}^{\left(m\right)}|o_{t}^{\left(m\right)})$;7:        Get the reward $r_{t}^{\left(m\right)}$ and the next observation $o_{t+1}^{\left(m\right)}$;8:     **end for**9:   **end for**10:   Get trajectory $\tau ={\left\{o_{t},a_{t},r_{t}\right\}}_{t=1}^{T}$;11:   Compute the cumulative discounted reward $R_{t}$, the state-value function $V_{\phi_{old}}$, and the advantage ${\left\{A_{t}^{\left(m\right)}\right\}}_{t=1}^{T}$ (According to (24)).12:   Split τ, $R_{t}$, and $A_{t}$ into chunks of length $N_{b}$, and store them in *U*.13:   **for** mini-batch j=1,...,Bch **do**14:     Randomly select a chunk from *U* as mini-batch *b*;15:     Compute gradients of (25) and (26) on θ and ϕ, respectively, using mini-batch *b*;16:     Update θ and ϕ using by Adam.17:   **end for**18:**end for**

### 3.3. CF-MAB-Based Scheme for Long-Term Consumption Minimization

The deployment and training of neural networks as agents can introduce high requirements for hardware and software in UDs. To reduce the overheads and complexity further, we propose a lightweight scheme that separates the association from the offloading decision and the computing resource allocation. The association directly determines the achievable rates between UDs and UAVs. Then, we find the closed-form offloading proportion and computing resource allocation for UDs given achievable rates. We first explain how to obtain the closed-form solution for the given association. Then, we explain the details of the MAB-based association. Finally, we summarize the CF-MAB-based scheme.

#### 3.3.1. Closed-Form Offloading and Computing Resource Allocation for a Given Association

For a given association, each UD decides its offloading ratio and computation resources to minimize its individual cost. Assuming that UD *m* is associated with UAV *n* in timeslot *t*, the problem at UD *m* can be formulated into

(31a) $$\begin{array}{ccc} P2:\; & \min_{\alpha_{m,t},f_{m,n,t}}C_{m,t} & \end{array}$$

(31b) $$\begin{array}{cc} s.t.\; & \alpha_{m,t}\in \left[0,1\right], \end{array}$$

(31c) $$\begin{array}{cc} & f_{m,n,t}\in \left[0,F_{\max}\right], \end{array}$$

(31d) $$\begin{array}{cc} & \tau_{m,t}\leq \tau^{\max}. \end{array}$$

Problem P2 is a short-term decision problem and can be solved at each UD independently.

From (31d), we can obtain

(32) $$\tau_{m,n,t}^{\mathrm{trans}}+\tau_{m,n,t}^{\mathrm{comp}}\leq \tau_{\max}$$

and

(33) $$\tau_{m,t}^{\mathrm{local}}\leq \tau_{\max}.$$

Substituting (4), (6), and (8) into (32) and (33), respectively, and combining (31c), we can obtain

(34) $$1-\frac{f_{u}\tau_{\max}}{D_{m,t}s}\leq \alpha_{m,t}\leq \frac{\tau_{\max}}{\frac{D_{m,t}}{r_{m,n,t}}+\frac{D_{m,t}s}{F_{\max}}}.$$

Usually, the system should satisfy $\frac{\tau_{\max}}{\frac{D_{m,t}}{r_{m,n,t}}+\frac{D_{m,t}s}{F_{\max}}}\leq 1$ so that all tasks can be executed at the MEC server. We can also obtain

(35) $$\frac{\alpha_{m,t}D_{m,t}s}{\tau_{\max}-\frac{\alpha_{m,t}D_{m,t}}{r_{m,n,t}}}\leq f_{m,n,t}\leq F_{\max}.$$

**Figure 2:**
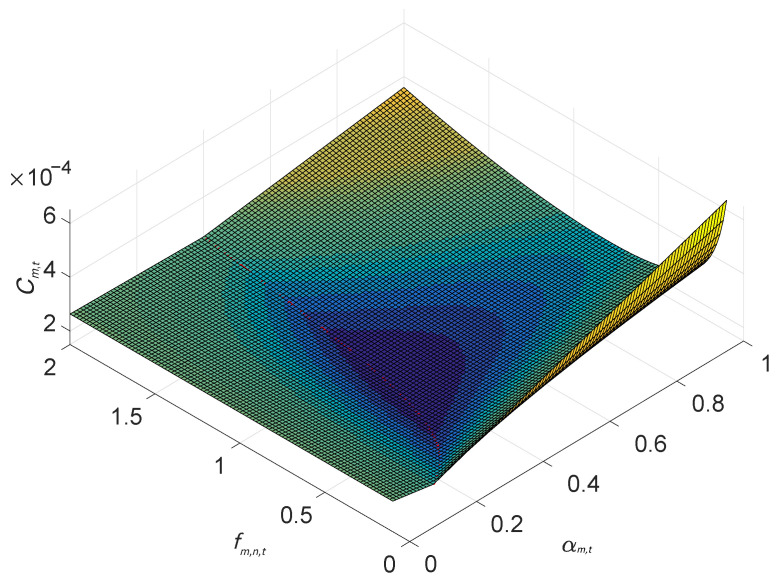
Plot of individual cost function $C_{m,t}$ with given association.

We further analyze the individual cost function and obtain

(36) $$C_{m,t}=\left\{\begin{array}{c} \lambda (E_{m,t}^{\mathrm{local}}+E_{m,n,t}^{\mathrm{trans}}+E_{m,n,t}^{\mathrm{comp}})+\left(1-\lambda \right)(\tau_{m,n,t}^{\mathrm{trans}}+\tau_{m,n,t}^{\mathrm{comp}}),\alpha_{m,t}\geq \alpha_{m,t}^{★}\\ \lambda (E_{m,t}^{\mathrm{local}}+E_{m,n,t}^{\mathrm{trans}}+E_{m,n,t}^{\mathrm{comp}})+\left(1-\lambda \right)\tau_{m,t}^{\mathrm{local}},\alpha_{m,t}<\alpha_{m,t}^{★} \end{array}\right.$$

where $\alpha_{m,t}^{★}=\frac{\frac{s}{f_{u}}}{\frac{1-\alpha_{m,t}}{f_{u}s}-\frac{\alpha_{m,t}}{r_{m,n,t}}}$ is obtained by setting $\tau_{m,n,t}^{\mathrm{trans}}+\tau_{m,n,t}^{\mathrm{comp}}=\tau_{m,t}^{\mathrm{local}}$. We check the gradient of $C_{m,t}$ on $\left\{\alpha_{m,t},f_{m,n,t}\right\}$, i.e., $\nabla_{\alpha_{m,t},f_{m,n,t}}C_{m,t}$, and find no stationary points in the feasible region. Consequently, the minimum value of $C_{m,t}$ is located at the boundaries with (1) $\alpha_{m,t}=1$, (2) $f_{m,n,t}=F_{max}$, and (3) $\alpha_{m,t}=\alpha_{m,t}^{★}$. A typical case of $C_{m,t}$ is shown in Figure 2.

When $\alpha_{m,t}=1$, UD *m* offloads all its tasks to the MEC server. The cost function becomes

(37) $$C_{m,t}=\lambda \kappa_{1}D_{m,t}sf_{m,n,t}^{2}+\frac{\left(1-\lambda \right)D_{m,t}s}{f_{m,n,t}}+\frac{\lambda PD_{m,t}+\left(1-\lambda \right)D_{m,t}}{r_{m,n,t}}.$$

To achieve the minimum cost, we set $\frac{dC_{m,t}}{df_{m,n,t}}=0$ and obtain the optimal solution $f_{m,n,t}^{*}=\sqrt[{3}]{\frac{1-\lambda }{2\lambda \kappa_{1}}}$. We also get the lower bound $f_{m,n,t}^{\mathrm{lb}}=\frac{D_{m,t}s}{\tau_{\max}-\frac{D_{m,t}}{r_{m,n,t}}}$ from (35). Then, we obtain a optimal solution candidate $\left(1,\min\{f_{m,n,t}^{*},f_{m,n,t}^{lb}\}\right)$.

When $f_{m,n,t}=F_{\max}$, $C_{m,t}$ is a piecewise linear function with respect to $\alpha_{m,t}$. It is monotonically increasing with $\alpha_{m,t}\geq \alpha_{m,t}^{★}$. Moreover, if $\lambda <\frac{1}{\kappa_{1}f_{u}F_{\max}^{2}-\kappa_{2}f_{u}^{3}+1}$ and $r_{m,n,t}\geq \frac{\lambda Pf_{u}}{10\lambda f_{u}^{3}+\left(1-\lambda \right)-\lambda F_{\max}^{2}f_{u}}$, $C_{m,t}$ is monotonically decreasing with $\alpha_{m,t}\leq \alpha_{m,t}^{★}$. Then, the optimal candidate is $\left(\alpha_{m,t}^{★},F_{\max}\right)$. Otherwise, $C_{m,t}$ is monotonically increasing with $\alpha_{i}\leq \alpha_{m,t}^{★}$. Then, we get the lower bound $\alpha_{m,t}^{lb}=1-\frac{f_{u}\tau_{\max}}{D_{m,t}s}$ from (34) and the optimal candidate is $\left(\alpha_{m,t}^{lb},F_{\max}\right)$.

When $\alpha m,t=\alpha_{m,t}^{★}$, the cost function $C_{m,t}$ becomes

(38) $$C_{m,t}=\frac{f_{m,n,t}}{\left(\frac{f_{u}}{r_{m,n,t}s}+1\right)f_{m,n,t}+f_{u}}\left(\lambda \kappa_{1}f_{m,n,t}^{2}+\frac{\lambda P}{r_{m,n,t}s}-\lambda \kappa_{2}f_{u}^{2}-\frac{1-\lambda }{f_{u}}\right)D_{m,t}s+\left(\lambda \kappa_{2}f_{u}^{2}+\frac{1-\lambda }{f_{u}}\right)D_{m,t}s.$$

We set $\frac{dC_{m,t}}{df_{m,n,t}}=0$ and obtain

(39) $$f_{m,n,t}^{★}=\frac{X-3\sqrt[{3}]{2}\kappa_{1}f_{u}}{6\sqrt[{3}]{2}\kappa_{1}\left(\frac{f_{u}}{r_{m,n,t}}s+1\right)}+\frac{3f_{u}^{2}\kappa_{1}^{2}}{2^{\frac{2}{3}}\kappa_{1}\left(\frac{f_{u}}{r_{m,n,t}s}+1\right)X}$$

where $X={\left(Y+\sqrt{Y^{2}-2916f_{u}^{6}\kappa_{1}^{6}}\right)}^{\frac{1}{3}}$, $Y=f_{u}^{3}\kappa_{2}Z-\frac{f_{u}PZ}{r_{m,n,t}s}+Z-54f_{u}^{3}\kappa_{1}^{3}$, and $Z=108\kappa_{1}{\left(\frac{f_{u}}{r_{m,n,t}s}+1\right)}^{2}$. Then, we get a optimal candidate $\left(\alpha_{m,t}^{★},f_{m,n,t}^{★}\right)$.

Finally, we can obtain the optimal offloading proportion $\alpha_{m,t}^{\mathrm{opt}}$ and computing resource allocation $f_{m,n,t}^{\mathrm{opt}}$ for each UD from the three candidates.

#### 3.3.2. MAB-Based Long-Term Association

We tackle the long-term association problem using a distributed MAB scheme. Each UD is an agent that selects its association to maximize its long-term achievable rate. The achievable rate of each UD depends on its channel gain and association with other UDs. Then, it varies over time due to the UAVs moving and associations changing. On the other hand, a slight variety will incur frequent handover, especially when a UD can associate with different UAVs to achieve similar rates. Therefore, the MAB-based scheme should obtain the channel conditions in a timely manner and avoid frequent handover.

Each UD agent decides its association and receives a simultaneous reward at each timeslot. Then, it updates its exploration and exploitation strategy using the cumulative rewards. The weight, the association probability, and the reward function of UAV *n* for UD *m* in timeslot *t* are denoted by $w_{m,n,t}$, $p_{m,n,t}$, and $R_{m,n,t}$, respectively.

Each UD selects its association according to the *probability*

(40) $$p_{m,n,t}=\left(1-\gamma_{0}\right)\frac{w_{m,n,t}}{\sum_{n^{\prime }\in N}w_{i,n^{\prime },t}}+\frac{\gamma_{0}}{N}$$

where $\gamma_{0}\in \left(0,1\right)$ adjusts the proportion of exploitation and exploration.

After its action, each UD receives its reward and the reward function is defined as

(41) $$R_{m,n,t}=\left\{\begin{array}{c} \psi_{m,n,t}\tanh\left(r_{m,n,t}-{\bar{r}}_{m,n,t}-{\tilde{r}}_{m,n,t}\right),r_{i,j,t}\geq {\bar{r}}_{m,n,t}\\ 0,\mathrm{otherwise} \end{array}\right.$$

where tanh(·) is the hyperbolic tangent activation function; $\psi_{m,n,t}$ is a reward discount rate to reduce frequent handover expressed as

(42) $$\psi_{m,n,t}=\left\{\begin{array}{c} 1,a_{m,n,t}=a_{m,n,t-1}\\ \psi ,a_{m,n,t}\neq a_{m,n,t-1} \end{array}\right.$$

with the discount factor ψ∈[0,1]; ${\bar{r}}_{m,n,t}$ is the average achieved rate in the past *L* timeslots, expressed as

(43) $${\bar{r}}_{m,n,t}=\frac{1}{L}\sum_{\tau =t-L}^{\tau =t-1}r_{m,n,\tau };$$

and ${\tilde{r}}_{m,n,t}$ is the bias to balance the load of UAVs, expressed as

(44) $$\begin{array}{ccc} {\tilde{r}}_{m,n,t} & = & \frac{1}{\left|M_{n,t}\right|}\sum_{k\in M_{n,t}}r_{k,n,t}-\frac{1}{\left|M_{n,t}\right|-1}\sum_{k^{\prime }\in M_{n,t},k^{\prime }\neq m}r_{k^{\prime },n,t} \end{array}$$

where $M_{n,t}$ is the set of UDs that associate with UAV *n* at timeslot *t*.

Then, each UD can obtain its cumulative reward and update the weight as

(45) $$G_{m,n,t}=\sum_{\tau =1}^{\tau =t}\frac{R_{m,n,\tau }}{p_{m,n,\tau }},$$

and

(46) $$w_{m,n,t+1}=\exp\left(\eta G_{m,n,t}\right)=w_{m,n,t}\exp(\eta \frac{R_{m,n,t}}{p_{m,n,t}}),$$

respectively, where η>0 determines how aggressively the algorithm to learn and update the distribution.

**Figure 3:**
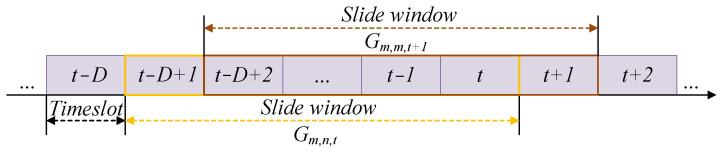
The slide window with length *D*.

It should be noted that the historical information becomes outdated and harmful for the current decision when the UAVs pass over a long distance. To improve the learning performance, we set a slide window with length *D* as shown in Figure 3, and the accumulative reward becomes the accumulation from the latest *D* timeslots as

(47) $$G_{m,n,t}=\sum_{\tau =t-D+1}^{\tau =t}\frac{R_{m,n,\tau }}{p_{m,n,\tau }}.$$

#### 3.3.3. CF-MAB-Based Algorithm

Finally, the CF-MAB-based algorithm at each UD is summarized in Algorithm 2. We focus on one UD and can find that each UD’s behavior is similar to the *EXP3* algorithm [52]. The convergence proof of the algorithm is given in our previous work [53].
**Algorithm** **2** CF-MAB-based algorithm**Require:** η, $\gamma_{0}$1:Initialize $G_{m,n,t}^{0}=0$, $w_{m,n,t}^{0}=1$ for m∈M and n∈N;2:**for** each timeslot t=1,…,T **do**3:   **for** each UD m∈M **do**4:     Calculate probability distribution $p_{m,n,t}$ with (40);5:     Make action $a_{m,n,t}$ randomly according to the probabilities $\left\{p_{m,1,t},\ldots ,p_{i,N,t}\right\}$;6:     Calculate the achieved rate $r_{m,n,t}$;7:     Obtain the optimal offloading proportion $\alpha_{m,t}^{\mathrm{opt}}$, computing resource allocation and $f_{m,n,t}^{\mathrm{opt}}$, and the minimum cost $C_{m,t}\left(\alpha_{m,t}^{\mathrm{opt}},f_{m,n,t}^{\mathrm{opt}}\right)$;8:     Calculate rewards $R_{m,n,t}$ with (41) and cumulative rewards $G_{m,n,t}$ with (45) or (47);9:     Update the weights $w_{m,n,t+1}$;10:   **end for**11:**end for**

## 4. Performance Evaluation

**Figure 4:**
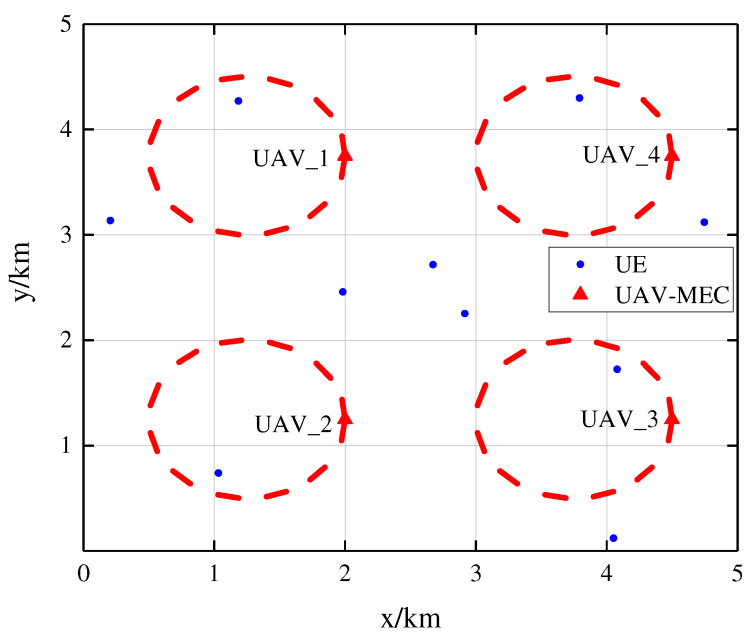
The locations of UAVs and UDs.

This section evaluates the proposed algorithms. As shown in Figure 4, we assume that M=10 UDs are randomly distributed in a 5×5 km^2^ square area with fixed locations and N=4 UAV-BSs are deployed above the square area with a fixed height of 300 m. The square area is divided into N=4 parts and each part shares its center with a UAV’s circular trajectory. The UAVs fly with a constant speed of 12.69 m/s and the trajectory has a radius of 0.75 km.

Some key parameters are given in the following. The channel coherence time is 5 ms and the channel state is constant during coherence time. The duration of each timeslot is 1 ms. The maximum latency is set to be the timeslot duration, i.e., $\tau_{\max}=1$ ms. The bandwidth is B=10 MHz, the noise power density is −174 dBm/Hz, and the transmit power of UD is P=0.35 W. The number of CPU cycles required to compute each bit is $s=10^{3}$ cycles/bit. The computing capability of each UD is $f_{u}=0.6$ GHz. The parameters that adjust energy consumption at the UAV server and at the UD are set to be $\kappa_{1}=10^{-27}$ and $\kappa_{2}=10^{-26}$, respectively. The parameter that balances latency and energy consumption is set to be λ=0.5. Unless otherwise stated, the total observed duration spans $5\times 10^{4}$ timeslots, the maximum computing resource is $F_{\max}=1.5$ GHz, and the volume of tasks that should be computed in one timeslot is $D_{t}=\sum_{m=1}^{M}D_{m,t}=10$ kbits.

To compare the performance, we employ MAPPO, received signal strength (RSS)-based, and random association strategies, and local-only, remote-only, closed-form, and random offloading strategies as benchmarks. Specifically,

- *CF-MAPPO* scheme: The UDs’ association is determined via a simplified MAPPO-based scheme to maximize the total achievable rate. Then, the offloading and computing resource allocation are obtained through a closed-form solution;
- *RSS + Remote-only* scheme: Each UD associates to the UAV with maximum RSS and all its tasks are executed at the UAV server;
- *Local-only* scheme: Each UD computes its tasks locally;
- *Random + Random* means: Each UD associates with a random UAV and a random part of its tasks are executed at the UAV server.

**Figure 5:**
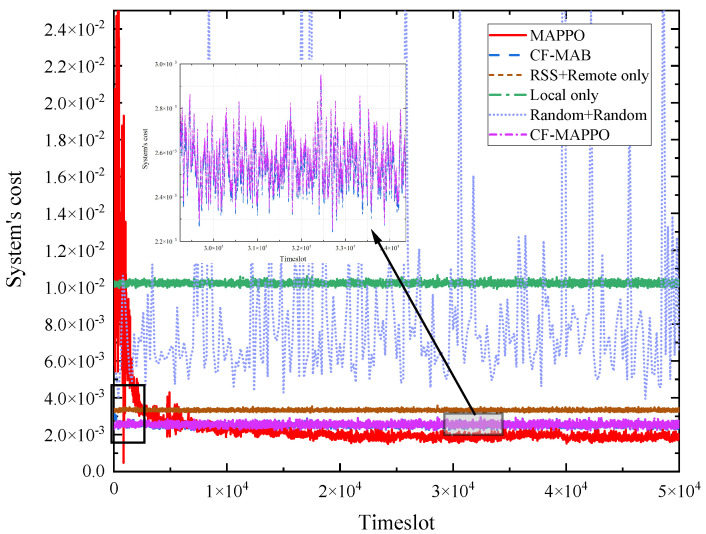
Convergence performance in the training stage.

Figure 5 compares the convergence performance in the training stage of the proposed MAPPO-based and CF-MAB-based schemes with benchmarks. It is obvious that the RSS + Remote-only scheme, the local-only scheme, and the Random + Random scheme are too simple to achieve satisfying performance. It can be found that the MAPPO-based scheme reduces the system cost significantly and converges to the lowest cost. The reason for this is that the MAPPO-based scheme can learn knowledge from the environment and the historic strategies effectively and efficiently. Then, all UDs can make satisfactory decisions to obtain the best performance. It can also be found that the CF-MAB-based scheme converges quickly and achieves sufficiently low system cost. The reason for this is that the CF-MAB-based scheme reduces the dimensions of the action space significantly by separating the association from the offloading proportion and computing resource allocation. The MAPPO-based scheme adopts centralized training using global information and can learn knowledge more sufficiently and effectively than CF-MAB-based scheme in which each UD only learns from individual historic information. The CF-MAPPO-based scheme achieves a slightly weaker performance than the CF-MAB-based scheme since they both decouple the joint optimization and use the closed-form resource allocation. Moreover, the CF-MAPPO-based scheme uses more effective centralized learning than the CF-MAB-based scheme, meaning that it converges faster.

**Figure 6:**
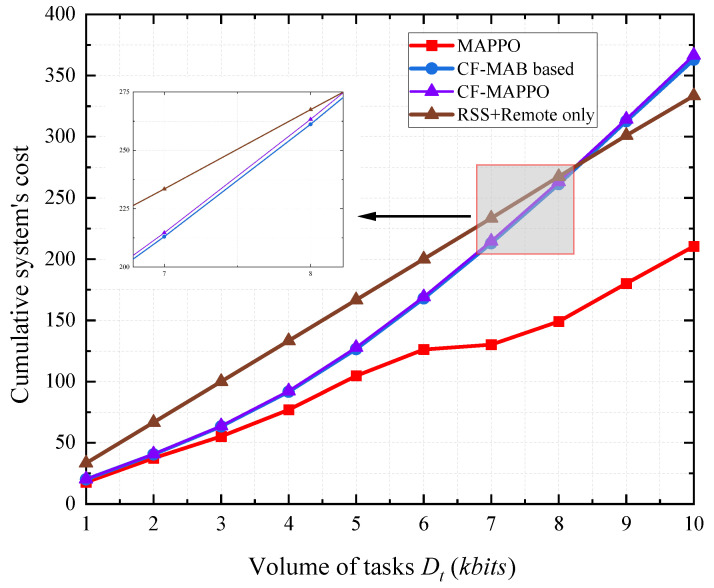
System’s cost with different task volumes.

Figure 6 shows the cumulative system cost with different task computing demands. The tasks may not be completed under the maximum constraints, in which case a failure happens. To show the performance of all schemes completely, we still count the system cost in failures. Both the latency and the energy consumption increase with the task volume, meaning that the cumulative system cost increases in all schemes. The MAPPO-based scheme outperforms the others under all task demands. The CF-MAB-based scheme and the CF-MAPPO-based scheme achieve a similar performance, while the CF-MAB-based scheme demonstrates marginally better performance. It can also be noted that the RSS + Remote-only scheme obtains lower cumulative system cost than the CF-MAB- and CF-MAPPO-based schemes with tasks ($D_{t}<8$ kbits). This is because the CF-MAB- and CF-MAPPO-based schemes take the latency constraints into consideration, meaning that they consume more energy during computing.

**Figure 7:**
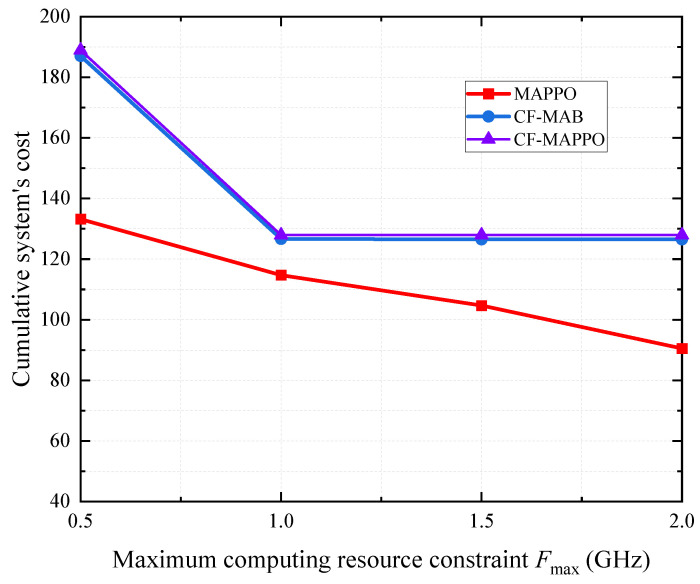
System’s cost under different computing resource constraints.

Figure 7 shows the cumulative system cost under different computing resource constraints. The volume of tasks in each timeslot is $D_{t}=5$ kbits. All schemes exhibit a monotonically decreasing cumulative system cost with respect to the computing resources below saturation thresholds (about 1 GHz). By optimally leveraging local and remote computation, these schemes achieve energy–latency trade-off optimization, resulting in a low cumulative system cost. The CF-MAB- and CF-MAPPO-based schemes decouple the transmission and the computation so that they cannot further reduce the cost when the computing resources are sufficiently large. The MAPPO-based scheme can use computation resources to compensate for communication in the joint optimization, meaning that it can further lower the cost.

**Figure 8:**
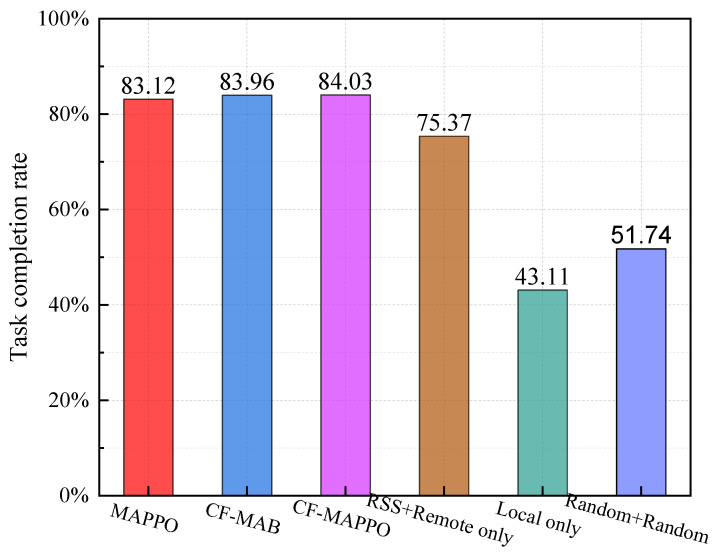
Task completion rate of different schemes.

The computation is completed successfully if the tasks are executed in the required time. Figure 8 shows the task completion rates of different schemes. The MAPPO-based scheme, the CF-MAB-based scheme, and the CF-MAPPO-based scheme outperform the other benchmarks significantly. Since the closed-form-based method takes the latency constraint into consideration, the CF-MAB and CF-MAPPO-based scheme can avoid failures and achieve slightly higher completion rates. The MAPPO-based scheme considers the latency constraint in the reward function and may select actions that result in exceeding latency, meaning that this scheme has a slightly lower completion rate. We further check Figure 6 and Figure 8 and can find that the RSS + Remote-only scheme has a significantly lower completion rate, though it achieves a slightly lower system cost.

## 5. Conclusions

This paper investigated the joint association, offloading, and computing resource allocation in multiple-UAV-assisted MEC networks. We introduce the cost function as a metric to balance the latency and the energy consumption. The joint latency and energy consumption minimization is transferred into a long-term cost minimization problem. Then, we adopt a multi-agent DRL framework and propose a MAPPO-based scheme and a CF-MAB-based scheme to solve this problem. The MAPPO-based scheme uses global observation for centralized learning and partial observation for decentralized execution. The CF-MAB-based scheme obtains associations to maximize the long-term transmission rates and achieve closed-form offloading and computing resource allocation. The numerical results validate the proposed schemes and show their superiority. Moreover, the MAPPO-based scheme can provide collaborative decisions for agents to obtain high performance in complex and dynamic environments. The CF-MAB-based scheme provides independent decisions and is suitable for scenarios needing rapid responses.

## Figures and Tables

**Table 1:** Comparison of related works.

Work	Objective			Method	
	Energy	Latency			
[11,12,13,14,15,16]	✓		short-term	Convex Optimization	
[17,18,19,20,21,22]		✓			
[23,24,25,26,27,28,29]	✓	✓			
[30,31]	✓		long-term	Lyapunov	
[32,33]	✓	✓			
[34]	Service quantity				
[35]		✓		AC	Single agent
[36]	✓	✓		DDQN	
[37]	✓	✓		DDPG	
[38]		✓		PPO	
[39,42]	✓	✓		MADDPG	Multiple agents
[40]	✓				
[41]		✓			
[43]	Task amount			MAPPO	
[44]	Energy efficiency				
[45]		✓			

**Table 2:** List of key notations.

Notation	Description
$\alpha_{m,t}$	Offloading task proportion of UD *m* at timeslot *t*
$\beta_{L}$, $\beta_{N}$	Path loss exponent for LOS and NLOS links
γ	Discount factor for rewards
$\gamma_{0}$	Parameter that adjusts the exploitation and exploration
$\gamma_{m,n,t}$	SNR between UD *m* and UAV *n* in timeslot *t*
δ	TD residual to calculate GAE in (24)
ϵ	Determines the interval of clip function
η	Parameter that determines how aggressively to learn and update
θ	Parameter of actor network
$\kappa_{1}$, $\kappa_{2}$	Weights of energy consumption by UAVs and UDs
$\kappa_{\theta ,i}^{\left(m\right)}$	Probability ratio between the current and the updated policy
λ	Adjusts the weights of energy consumption and latency
$\lambda_{0}$	Balances the bias and variance in GAE
$\mu_{L}$, $\mu_{N}$	Attenuation factor for LOS and NLOS links
$\pi^{\left(m\right)}$	Policy of agent *m*
τ	Latency
ϕ	Parameter of critic network
$a_{m,n,t}$	Association between UD *m* and UAV *n* in timeslot *t*
$a_{t}^{\left(m\right)}$	Action of agent *m* in timeslot *t*
$C_{t}$, $C_{m,t}$	System’s cost and individual cost in timeslot *t*
$D_{m,t}$	Volume of tasks
*E*	Energy consumption
$f_{u}$, $f_{m,n,t}$	Computing frequencies at UD and UAV
$o_{t}^{\left(m\right)}$	Observation of UD *m* at timeslot *t*
$r_{m,n,t}$	Achievable rate between UD *m* and UAV *n* in timeslot *t*
$r_{t}^{\left(m\right)}$	Reward of UD *m* at timeslot *t*
$R_{m,t}$	Individual reward function of UD *m* at timeslot *t*
$R_{M,t}$	System’s reward function at timeslot *t*

## Data Availability

The original contributions presented in this study are included in the article. Further inquiries can be directed to the corresponding author.
